# College students’ cognition and attitude toward medical knowledge education after a global public health event

**DOI:** 10.3389/fpubh.2025.1606919

**Published:** 2025-07-25

**Authors:** Wei Xiang, Hui Cao, Juan Wu, Peiru Chen, Qiong Chen, Fenghua Tao, Ting Jiang

**Affiliations:** ^1^Department of Orthopedics, Renmin Hospital of Wuhan University, Wuhan University, Wuhan, China; ^2^School of Law and Public Administration, Huaihua University, Huaihua, China; ^3^Department of Pediatrics ICU, Union Hospital, Tongji Medical College, Huazhong University of Science and Technology, Wuhan, China; ^4^Department of Nursing, Wuhan University Hospital, Wuhan University, Wuhan, China; ^5^Department of Neurological Rehabilitation, Zhongnan Hospital of Wuhan University, Wuhan, China

**Keywords:** medical knowledge education, public health event, cognition and attitude, medical and non-medical students, practical medical skills

## Abstract

**Introduction:**

The past global public health event has heightened governmental and societal awareness of the importance of health and medical knowledge education. This study aims to investigate and compare the attitude and cognition of non-medical students and medical students toward medical knowledge education.

**Methods:**

A self-administered, anonymous questionnaire was voluntarily completed by 304 university students recruited through the Wen-Juan-Xing online platform via WeChat QR codes or website links. The survey assessed participants’ cognition and attitudes regarding medical education.

**Results and discussion:**

Results indicated that the vast majority of students recognized the importance of promoting medical knowledge education to disseminate practical medical skills and health knowledge. Medical students demonstrated significantly deeper understanding of general medical knowledge and greater proficiency in practical medical skills compared to non-medical students. Among non-medical students, 63.4% identified the lack of dedicated medical curricula and training as the primary barrier to their learning of medical knowledge, while only 53.4% expressed satisfaction with their institution’s current medical education offerings. These findings underscore the critical role of medical knowledge education in enhancing public health literacy by disseminating general medical knowledge and practical skills. Non-medical institutions should prioritize medical education reforms, including innovative medical curricular designs and teaching methodologies, to better align with both student and societal demands for healthcare competency.

## 1 Introduction

Global public health events represent persistent challenges for humanity in the future (1). The previous COVID-19 pandemic persisted for over 3 years and caused numerous serious illness and deaths all over the world (2). Given the continuous viral mutations, some infectious diseases including coronavirus and the influenza epidemic currently still pose substantial threats to human health (3). Global public health event always caused serious challenges to medical staff, patients and the whole community population (4). To date, these diseases have not disappeared from our planet. In order to prevent these health disasters, people were once informed to protect themselves by proper mask-wearing, maintaining social distance, Observing respiratory hygiene etiquette, frequent hand hygiene, vaccination uptake, home confinement and self-isolation (5, 6). Public awareness regarding the severe consequences of these infectious diseases has markedly increased, and people are becoming increasingly accustomed to these lifestyle changes (7).

Global engagement plays a important role in mitigating and preventing the transmission of infectious diseases (8). The participation of community residents is crucial for effective epidemic containment (9). Evidence demonstrates that community-based interventions including vaccination programs, proper mask-wearing and public health education yield significant societal benefits (10). Previous study has shown that residents’ adequate knowledge and positive attitudes positively correlated with improved infectious diseases prevention and contol in their community (11). Notably, younger individuals with higher education were knowledgeable and showed positive attitudes toward infectious diseases (12). These studies reveal that the implementing of medical knowledge education program can enhance community resilience against disease outbreaks. In China, health literacy education is a fundamental component to achieve the goal of “Healthy China Initiative” (13). The contents of health literacy mainly include the knowledge of emergency rescue and practical medical skills, infectious diseases prevention and control, mental health management and healthy lifestyle promotion (13). Following the COVID-19 pandemic, people have begun to place greater value on and reflect more deeply about various health-related topics, including life and health, disease prevention, psychological well-being, and rehabilitation (7). It is therefore of great value to promote health in society by strengthening medical knowledge education.

As representatives of young residents, college students’ cognition and attitude toward health and medical knowledge education are closely associated with the future development of national healthcare policies and services (14). To date, the cognition and attitude of college students about general medical knowledge education after COVID-19 outbreak are seldom reported in literatures. It is of great significance for schools to gain information about the current attitudes and educational needs of students toward medical knowledge. In this study, we investigated the cognition and attitude of college students toward general medical knowledge among medical students and non-medical students. The results will provide valuable evidence to inform policy-making and help educational administrators take effective measures to enhance college students’ medical literacy, particularly in addressing public health events.

## 2 Method

### 2.1 Study design

This questionnaire was designed by teachers, doctors and medical scientists. This study was a cross-section study and took the form of multiple-choice questions to assess the cognition and attitude of college students toward the general medical knowledge. The contents of this questionnaire contained four sections: (1) demographic characteristics; (2) cognition about general medical knowledge; (3) attitude about medical knowledge education. Prior to implementation, the questionnaire underwent pilot testing, with subsequent revisions and refinements made based on identified issues and feedback. This study was an anonymous online survey with no collection of personally identifiable information and subjective content.

### 2.2 Data collection

This questionnaire was designed in Chinese and edited on the online survey platform Wen-Juan-Xing (www.wjx.cn). A QR code was generated and disseminated to college students via WeChat. These students were selected through stratified and staged random sampling. They are from 7 universities in different region of China, such as Wuhan University, Huazhong University of Science and Technology, Huaihua University, Yangtze University, South China University of Technology, Hubei Polytechnic Institute and Wuhan Institute of Technology. Participation in this study was entirely voluntary for all students, regardless of their medical or non-medical background. Participants accessed the questionnaire by scanning a WeChat QR code and completing the survey online. Repeated submission from the same IP address was prohibited and response time to complete the questionnaire of less than 90 s was considered invalid. The data was collected via the online survey platform from November 28th to December 4th, 2024.

### 2.3 Data analysis

Data analysis were performed by using of SPSS 20.0 software. Chi-square test and Fisher exact test (expected frequency<5) were used to analyze the data between medical students group and non-medical students group. A *p*-value of 0.05 or less is considered statistically significant.

## 3 Results

### 3.1 The demographic characteristics of the participants

This study used an online questionnaire survey and 304 valid responses were collected. The participants were recruited from 7 different universities in China. The medical students cohort comprised individuals majoring in clinical medicine, preventive medicine and nursing, while non-medical participants represented various fields including sociology, engineering, education, management, etc. Junior college students, undergraduate and graduate students were enrolled in this online questionnaire study. Among these 304 students, 126 males and 178 females were included. Of these participants, 143 (47.04%) students were in the medical students group and 161 (52.96%) students were in the non-medical students group. There was no significant difference in gender, nationality, region and parents’ education level among these participants (*p* > 0.05). The proportion of graduate participants among medical students was higher than that of non-medical students, because medical students in China always studied at a high level (*p* < 0.05). The complete demographic characteristics for both groups were listed in Table 1.

**Table 1:** The general demographic characteristics of the participants.

Demographic characteristics	Medical students Number proportion		Non-medical students Number proportion		*χ* ^2^	*p* value
Gender						
Men	52	36.36%	74	45.96%	2.875	0.09
Women	91	63.64%	87	54.04%		
Nationality						
Han	134	93.71%	151	93.79%	0.001	0.976
Minority	9	6.29%	10	6.21%		
Area						
Town	71	49.65%	70	43.49%	1.160	0.281
Country	72	50.35%	91	56.52%		
Grade						
Graduate students	42	29.37%	28	17.39%	24.324	<0.001^**^
Undergraduate students	82	57.34%	130	80.75%		
Junior students	19	13.29%	3	1.86%		
The highest educational level of parents						
College or above	28	19.58%	43	26.71%	3.712	0.294
High-school	45	31.47%	37	22.98%		
Junior high-school	55	38.46%	63	39.13%		
Primary school	15	10.49%	18	11.18%		

^**^Chi-square test for difference between medical students and non-medical students (*p* < 0.01 considered highly statistically significant).

### 3.2 Participants’ cognition about the general medical knowledge

As is shown in Table 2, medical students’ cognition of general medical knowledge was better than that of the non-medical students. The participants who have received medical training displayed superior understanding of practical medical skills, including cardiopulmonary resuscitation (CPR), automated external defibrillator (AED), surgical hand washing, and Heimlich maneuver. However, proficiency rates among non-medical students for these skills were 42.7, 24.8, 58.4 and 41%, respectively. The proportions were significantly lower than that of the medical students group (83.2, 60.1, 95.1, and 79%, respectively, *p* < 0.05). In response to the question: “What medical knowledge are you interested in?” More than half of the participants in both groups were interested in the areas of first aid (medical students: 79%, non-medical students: 73.9%), sports health (medical students: 69.9%, non-medical students: 73.3%) and mental health (medical students: 59.4%, non-medical students: 57.1%). In addition, medical students were more interested in some professional medical knowledge, such as medical ethics, chronic diseases, anatomy, sexual and reproductive health, etc. The results showed statistical significance between these two groups (*p* < 0.05). In response to the question: “What kinds of first aid skills have you mastered?” More than half of these participants believed that they were proficiency in wounds management (medical students: 80.4%, non-medical students: 57.1%). But in general, the proportions of students in both two groups who mastered these first aid skills were not high. In contrast, participants in medical student group demonstrated superior performance in these practical skills than non-medical students, including wounds management, external fixation of bone fracture, and immobilization and transfer of spine fracture (*p* < 0.05). Notably, 6.3% of medical students and 16.1% of non-medical students said that they did not master any first aid skills mentioned above (*p* < 0.05). These results indicated that medical students had a deeper understanding about the general medical knowledge.

**Table 2:** Participants’ cognition about general medical knowledge.

Participant’s cognition	Medical students Number Proportion		Non-medical students Number Proportion		*χ* ^2^	*p* value
Do you know the procedure of cardiopulmonary resuscitation (CPR)?						
Yes	119	83.2%	76	47.2%	42.704	<0.001^**^
No	24	16.8%	85	52.8%		
Do you know the procedure of surgical hand washing?						
Yes	136	95.1%	94	58.4%	55.447	<0.001^**^
No	7	4.9%	67	41.6%		
Do you know how to use the automated external defibrillator (AED)?						
Yes	86	60.1%	40	24.8%	38.875	<0.001^**^
No	57	39.9%	121	75.2%		
Do you know the procedure of Heimlich maneuver?						
Yes	113	79.0%	66	41.0%	45.234	<0.001^**^
No	30	21.0%	95	59.0%		
What medical knowledge are you interested in?						
Knowledge of first aid						
Yes	113	79.0%	119	73.9%	1.093	0.296
No	30	21.0%	42	26.1%		
Medical ethics						
Yes	59	41.3%	43	26.7%	7.192	0.007^**^
No	84	58.7%	118	73.7%		
Sports health						
Yes	100	69.9%	118	73.3%	0.422	0.516
No	43	30.1%	43	26.7%		
The treatment for chronic diseases						
Yes	76	53.1%	50	31.1%	15.229	<0.001^**^
No	67	46.9%	111	68.9%		
Anatomy						
Yes	69	48.3%	57	35.4%	5.151	0.023^*^
No	74	51.7%	104	64.6%		
The treatment for infectious diseases						
Yes	68	47.6%	68	42.2%	0.866	0.352
No	75	52.4%	93	57.8%		
Mental health						
Yes	85	59.4%	92	57.1%	0.164	0.685
No	58	40.6%	69	42.9%		
Sexual and reproductive health						
Yes	84	58.7%	45	28.0%	29.394	<0.001^**^
No	59	41.3%	116	72.0%		
Traditional Chinese medicine						
Yes	65	45.5%	58	36.0%	2.795	0.095
No	78	54.5%	103	64.0%		
Medical Interdisciplinary						
Yes	38	26.6%	22	13.7%	7.967	0.005^**^
No	105	73.4%	139	86.3%		
What first aid skills do you have mastered?						
Wound management						
Yes	115	80.4%	92	57.1%	18.886	<0.001^**^
No	28	19.6%	69	42.9%		
External fixation of limb fractures						
Yes	75	52.4%	24	14.9%	48.602	<0.001^**^
No	68	47.6%	137	85.1%		
Immobilization and transfer of spinal fractures						
Yes	74	51.7%	16	9.9%	63.525	<0.001^**^
No	69	48.3%	145	90.1%		
Treat sunstroke						
Yes	77	53.8%	77	47.8%	1.098	0.295
No	66	46.2%	84	52.2%		
Save drowning						
Yes	68	47.6%	62	38.5%	2.530	0.112
No	75	52.4%	99	61.5%		
Save carbon monoxide poisoning						
Yes	44	30.8%	39	24.2%	1.635	0.201
No	99	69.2%	122	75.8%		
Treat burns and scalds						
Yes	59	41.3%	62	38.5%	0.239	0.625
No	84	58.7%	99	61.5%		
None of the above						
Yes	9	6.3%	26	16.1%	7.220	0.007^**^
No	134	93.7%	135	83.9%		

^*^Chi-square test for difference between medical students and non-medical students (*p* < 0.05 considered statistically significant).

^**^Chi-square test for difference between medical students and non-medical students (*p* < 0.01 considered highly statistically significant).

### 3.3 Participants’ attitude about the medical knowledge education

Table 3 presents participants’ attitudes toward medical knowledge education. Nearly all participants recognized the importance and necessity of promoting medical education, and more than half of the students in each group believed that they had some medical knowledge (medical students:94.4%, non-medical students:65.8%). However, 34.2% of non-medical students reported limited medical knowledge (*p* < 0.05). Additionally, 23.6% of non-medical students considered medical knowledge less relevant to their professional development (*p* < 0.05). In response to the question: “What is the purpose of medical knowledge education?” Over 80% students in both groups believed that medical knowledge education was aimed to enhance practical medical skills, improve health knowledge, and promote health behaviors. Among the participants, there were differences in medical knowledge acquisition methods. Medical students acquired medical knowledge primarily through courses, lectures, television and video, and physician guidance. However, 82.6% of non-medical students acquired medical knowledge via the internet (*p* < 0.05). Additionally, the proportion of non-medical students who acquired knowledge through advice from friends or school was higher than that of medical students group (*p* < 0.05). In response to the question: “What are the main barriers for popularizing medical knowledge education?” 51.7% of medical students believed that the difficulties of medical course content and examination were the main obstacles to affect their learning (*p* > 0.05), while 63.4% of non-medical students believed that the limited courses and training availability was the major barrier (*p* < 0.05). Therefore, only 53.4% of non-medical students were satisfied with the current situation of medical knowledge education which was significantly lower than medical students (90.2%, *p* < 0.05). Notably, more than 90% of the students in both groups (medical students: 97.9%, non-medical students: 92.5%) were willing to participate in medical education courses or activities.

**Table 3:** Participants’ attitude about medical knowledge education.

Participant’s attidude	Medical students Number proportion		Non-medical students Number proportion		*χ^2^*	*p* value
Do you know anything about medical knowledge?						
A great many	28	19.6%	1	0.6%	59.363	<0.001^**^
Moderately	107	74.8%	105	65.2%		
Rarely	8	5.6%	55	34.2%		
Is it important to popularize medical knowledge education?						
Very important	108	75.5%	106	65.9%		0.078
Moderately important	35	24.5%	53	32.9%		
Unimportant	0	0	2	1.2%		
Is it important for your professional development to learn medical knowledge?						
Very important	112	78.3%	49	30.4%	79.475	<0.001^**^
Moderately important	31	21.7%	74	46.0%		
Unimportant	0	0	38	23.6%		
What is the purpose of medical knowledge education?						
Popularize the professional medical knowledge						
Yes	100	69.9%	85	52.8%	9.334	0.002^**^
No	43	30.1%	76	47.2%		
Popularize practical medical skills						
Yes	117	81.8%	148	91.9%	6.918	0.009^**^
No	26	18.2%	13	8.1%		
Popularize health knowledge and health behaviors						
Yes	122	85.3%	140	87.0%	0.171	0.679
No	21	14.7%	21	13.0%		
Promote the development of medical-related interdisciplinary fields						
Yes	89	62.2%	91	56.5%	1.025	0.311
No	54	37.8%	70	43.5%		
What is your approach to acquire medical knowledge?						
Self-study courses						
Yes	110	76.9%	53	32.9%	58.967	<0.001^**^
No	33	23.1%	108	67.1%		
Books, magazines and literatures						
Yes	83	58.0%	80	49.7%	2.125	0.145
No	60	42.0%	81	50.3%		
Browse internet resource						
Yes	100	69.9%	133	82.6%	6.801	0.009^**^
No	43	30.1%	28	17.4%		
Lectures, TV and video						
Yes	111	77.6%	88	54.7%	17.664	<0.001^**^
No	32	22.4%	73	45.3%		
Physician instruction						
Yes	98	68.5%	62	38.5%	27.38	<0.001^**^
No	45	31.5%	99	61.5%		
Consultation from friends or school						
Yes	45	31.5%	78	48.4%	9.063	0.003^**^
No	98	68.5%	83	51.6%		
What are the main obstacles to the popularization of medical knowledge education?						
The course content is boring and unappealing						
Yes	39	27.3%	58	36.0%	2.670	0.102
No	104	72.3%	103	64.0%		
The course content is not practical						
Yes	20	14.0%	18	11.2%	0.545	0.460
No	123	86.0%	143	88.8%		
The course content and examination are difficult						
Yes	74	51.7%	97	60.2%	2.224	0.136
No	69	48.3%	64	39.8%		
The course takes too much time.						
Yes	35	24.5%	55	34.2%	3.409	0.065
No	108	75.5%	106	65.8%		
The school does not provide relevant courses or trainings.						
Yes	60	42.0%	102	63.4%	13.928	<0.001^**^
No	83	58.0%	59	36.6%		
Are you willing to participate in medical education courses or activities?						
Yes	140	97.9%	149	92.5%	4.631	0.031^*^
No	3	2.1%	12	7.5%		
Are you satisfied with the current state of medical knowledge education in school?						
Yes	129	90.2%	86	53.4%	49.517	<0.001^**^
No	14	9.8%	75	46.6%		

^*^ Chi-square test for difference between medical students and non-medical students (*p* < 0.05 considered statistically significant).

^**^ Chi-square test for difference between medical students and non-medical students (*p* < 0.01 considered highly statistically significant).

## 4 Discussion

The COVID-19 pandemic, as a global public health event, has caused immeasurable harm on populations worldwide. This epidemic has claimed thousands of lives and caused lifelong complications to numerous survivors (15). This infectious disease has profound impacts on many aspects of our lives, including healthcare systems, mental health, education, economy, etc. (16–19). Confronted with this unprecedented crisis, we have recognized the critical importance of medical knowledge dissemination and initiated reevaluation of existing deficiencies in higher education (20). The aim of this study was to assess the cognition and attitude of medical knowledge education among both non-medical students and medical students. The results demonstrated that almost all the students recognized the importance of medical education, with strong consensus advocating for enhanced institutional promotion of medical knowledge in higher education.

Proficiency in practical medical skills can objectively reflect students’ perception of medical knowledge (21, 22). In this study, we found that medical students demonstrated significantly superior understanding of standardized procedures for these practical medical skills including CPR, AED, surgical hand washing and the Heimlich maneuver compared to non-medical students. As for some other first-aid skills, medical students demonstrated greater competence in these professional practical skills of wound management, limbs fracture fixation, and spinal fracture immobilization and transportation. These results indicate that medical knowledge education in medical colleges can provide students with more systematic training in both theoretical knowledge and practical skills than non-medical schools. The implementation of systematic and professional medical knowledge curriculum and training are essential to improve the practical medical skills of non-medical students (20). Notably, 16.1% of non-medical students reported no first-aid skills proficiency- a proportion significantly higher than medical students. The results suggest that some non-medical students pay less attention to medical knowledge and do not take the initiative to learn. Some non-medical colleges and universities do not pay enough attention to strengthening medical education in their teaching plans. Admittedly, college students’ participation in medical education will foster a positive sense of social responsibility, and promote practical medical knowledge dissemination (23). Enhancing medical literacy in higher education represents not only a vital strategy for disease prevention, but also an important step toward achieving the goal of “Healthy China Initiative” in the future.

Medical education is essential for disseminating medical knowledge and fostering a healthy lifestyle among the population (20, 23, 24). Our study demonstrates near-universal consensus among students regarding the importance of incorporating medical education in higher education. Most students agree that medical education plays an important role in promoting practical medical skills, health knowledge and health behaviors. With the development of life science, the medical-related interdisciplinary areas have also developed rapidly in recent years, which are projected to remain a key focus of future medical education (25). Increasing numbers of students recognize the growing significance of interdisciplinary medicine as a promising career pathway (26). However, more than 30% (34.2%) of non-medical students report limited medical knowledge - significantly higher than medical students. Furthermore, 23.6% of non-medical students perceive medical education as irrelevant to their professional development. This likely stems from insufficient medical curriculum in non-medical universities, leading students to underestimate its societal value in health promotion. Among the participants, both medical students and non-medical students have their own educational preferences. For example, most students are interested in first aid, sports health and mental health. However, medical students are more likely to be interested in some professional topics, including chronic disease management, sexual and reproductive health, medical ethics, and anatomy. Medical students have received more systematic and comprehensive medical education, so that they have a deeper understanding and more standardized skill acquisition than non-medical students. For these participants, there are obvious differences between medical students and non-medical students regarding the main ways of acquiring medical knowledge. Medical students have more extensive and varied opportunities to obtain medical knowledge, while non-medical students acquire medical knowledge predominantly relied on internet sources and informal advice from peers or school. The results suggest that medical students are more interested in medical knowledge, and exhibit greater intrinsic motivation to pursue professional medical knowledge. Crucially, formal education is still an indispensable avenue for non-medical students to acquire medical knowledge. Compared with Internet resources, school medical education can provide students with professional, standardized, evidence-based medical content. However, due to the lack of a comprehensive medical education system, non-medical colleges face greater difficulties in popularizing medical knowledge, including faculty shortages, medical curricular marginalization, pedagogical mismatch, etc. For non-medical students, the contents of medical courses are boring and misaligned with their core disciplines, and the exams in medical courses are challenging. These structural barriers warrant urgent institutional attention to optimize cross-disciplinary medical education.

The past global public health event has provided valuable insight and experience to medical education reform in China (24). This global public event increased collage students’ awareness and compliance with hygiene protocols (21). When confronting public health emergency, collective participation becomes imperative. College students are an important force in responding to public health emergencies. Strengthening their capacities to address public health crises represents a fundamental strategy for future epidemic prevention and control (20). Therefore, it is very important to assess students’ cognition and attitude about medical knowledge. Non-medical schools need to reconceptualize their pedagogical approaches. They should recognized the purpose of medical education is not to train medical specialists but to promote healthy lifestyles and practical medical skills. Non-medical schools should understand students’ needs for medical knowledge and adjust their educational programs and teaching methods to improve college students’ ability in dealing with public health emergencies. Additionally, non-medical institutions should implement innovative pedagogical approaches in medical curriculum design and diversify knowledge dissemination channels to develop a comprehensive medical education framework that can engage student learners effectively, align with societal healthcare demands, and bridge theoretical knowledge with practical applications.

Moreover, this study has several limitations. For instance, while WeChat QR codes enable convenient sampling, they may introduce selection bias. The reliance on self-reported data carries inherent risks of measurement bias. In the future, these research directions warrant further attention, including: longitudinal studies on medical literacy trends, evaluations of medical education interventions, and qualitative research on barriers to student engagement. Such studies would strengthen the theoretical and empirical foundations guiding medical education reform.

## 5 Conclusion

Medical knowledge education has a positive impact on promoting public health. The results of this study provide evidence for the implementation of practical medical knowledge education among college students in the post-pandemic era. To enhance preparedness for future global health crises, higher education institutions, especially non-medical colleges, need to reform their teaching programs, develop medical knowledge education plans, and implement practical medical skills training.

## Data Availability

The raw data supporting the conclusions of this article will be made available by the authors, without undue reservation.
